# The Relationship between Serum Concentrations of Pro- and Anti-Inflammatory Cytokines and Nutritional Status in Patients with Traumatic Head Injury in the Intensive Care Unit

**DOI:** 10.3390/medicina55080486

**Published:** 2019-08-15

**Authors:** Mohammed I. M. Gubari, Abdolreza Norouzy, Mostafa Hosseini, Fadhil A. Mohialdeen, Mohammad Javad Hosseinzadeh-Attar

**Affiliations:** 1 Department of Clinical Nutrition, School of Nutritional Sciences and Dietetic, Tehran University of Medical Sciences, Tehran 1416643931, Iran; 2 Department of Epidemiology and Biostatistics, School of Public Health, Tehran University of Medical Sciences, Tehran 1417613151, Iran; 3 Community Health Department, Technical College of health, Sulaimani Polytechnic University, Sulaimani 46001, Iraq; 4 Centre of Research Excellence in Translating, Nutritional Science to Good Health, The University of Adelaide, Adelaide, SA 5005, Australia

**Keywords:** pro- and anti-inflammatory cytokines, traumatic head injury (THI), lean body mass, fat body mass, APACHE II and SOFA scores

## Abstract

*Background and objective:* The aim of the present study was to examine the relationship between serum levels of pro-inflammatory cytokines (IL-6, IL-1β, and TNF-α) and anti-inflammatory cytokines (IL-10) measured once at the baseline with changes in nutritional status of patients with traumatic head injury (THI) assessed at three consecutive times (24 h after admission, day 6 and day 13) during hospital stay in the intensive care unit (ICU). *Materials and Methods:* Sixty-four patients with THI were recruited for the current study (over 10 months). The nutritional status of the patients was determined within 24 h after admission and on days 6 and 13, using actual body weight, body composition analysis, and anthropometric measurements. The APACHE II score and SOFA score were also assessed within 24 h of admission and on days 6 and 13 of patients staying in the ICU. Circulatory serum levels of cytokines (IL-6, IL-1β, TNF-α, and IL-10) were assessed once within 24 h of admission. *Results:* The current study found a significant reduction in BMI, FBM, LBM, MAUAC, and APM, of THI patients with high serum levels the cytokines, over the course of time from the baseline to day 7 and to day 13 in patients staying in the ICU (*p* < 0.001). It was also found that patients with low levels of some studied cytokines had significant improvement in their nutritional status and clinical outcomes in term of MAUAC, APM, APACHE II score and SOFA score (*p* < 0.001 to *p* < 0.01). *Conclusion:* THI patients who had high serum levels of studied cytokines were more prone to develop a reduction of nutritional status in terms of BMI, FBM, LBM MAUAC and APM over the course of time from patient admission until day 13 of ICU admission.

## 1. Introduction

It is essential to maintain the nutritional status of traumatic brain injury (TBI) patients in intensive care unit (ICU) to reduce morbidity and mortality. The chief risk factors for nutrition-related issues include the need for starting nutritional support and the level of disease-related catabolism [1]. A number of clinical indicators together with nutrition testing tools can be used to determine the nutritional status of seriously ill patients. The indicators include anthropometric measurements (e.g., body weight, body mass index (BMI), mid-arm muscle circumference (MAMC), triceps skinfold thickness (TSF), and calf circumference), body composition markers and biochemical indices (e.g., visceral protein, total protein, albumin, and prealbumin) [2]. However due to high level of inflammatory response in trauma patients, they might be prone to develop acute phase protein, in which the biochemical indices may not be a good indicator of nutritional status [3]. TBI patients have been reported to undergo numerous changes in metabolic, immunological, and physiological pathways [4]. Moreover, during their stay in the ICU, patients with trauma in general and traumatic brain injury (TBI) in particular experienced a wide range of major changes, including hypermetabolism and an increase in protein catabolism, increased energy expenditure, and insulin resistance and tolerance [5,6,7,8,9,10,11]. These changes might induce systemic inflammatory response syndrome (SIRS), which in turn ends in the release of endogenous cytokines that play a substantial role in producing further metabolic changes [12].

Interleukin 6 (IL-6) is a pro-inflammatory cytokines and has been referred to as one of the chief mediators of the acute-phase response, and there is abundant evidence showing that trauma, burns, surgery, sepsis, cardiogenic shock, and cancer are associated with an increase in the concentration of IL-6 [13,14,15]. Furthermore, various studies have indicated that an increased IL-6 concentration is correlated with the severity of disease and dysfunction in different organs [16,17,18,19,20,21,22,23]. In addition to IL-6, it has been reported that there is an increase in the concentration of tumor necrosis factor (TNF-α), another pro-inflammatory cytokine, during and after trauma. Moreover, increases in TNF-α and IL-6 have been referred to as indicators of the development of a nosocomial infection in patients with trauma [14]. As indicated in a recent study conducted by Battista et al. (2016), patients with TBI experience an increase in an amount of cytokines, such as TNF-α, which worsens the outcomes for such patients [24].

In addition to the above-mentioned interleukins (i.e., IL-6 and TNF), it has been reported that IL-1β, a pro-inflammatory cytokine, has a marked effect on trauma severity and mortality among TBI patients [25,26,27]. Research studies have also focused on IL-10 as an anti-inflammatory cytokine in patients with trauma and revealed that the level of IL-10 increases in the cerebral spinal fluid (CSF) and serum of patients with isolated, severe TBI [28]. Some studies have indicated that higher levels of IL-10 are associated with better outcomes. For instance, an early study indicated that there is a link between increased IL-10 levels in the CSF and mortality in children with TBI [29], and a more recent study focusing on adults with severe TBI revealed a similar association between increased IL-10 and mortality [30]. Studies have also revealed a higher concentration of IL-10 in the CSF of patients with an unfavorable outcome Glasgow outcome scale score (GOS < 4), which was assessed 6 months following the injury [31]. Nevertheless, no relationship between IL-10 and outcome has been reported by other studies [32,33]. In their study of children with TBI, Lo et al., (2010) measured serum IL-10 levels on day 1 and could not differentiate severe and non-severe injuries or predict favorable outcomes, even when compared with Glasgow coma scale score (GCS) [34]. The results of a more recent study indicated that there was no correlation between the level of IL-10 in the serum or CSF and the outcome assessed after 6 months using the Glasgow outcome scale extended (GOSE) score [35]. Additionally, IL-10 has been reported to be associated with in-hospital mortality in patients with severe traumatic brain injury. In addition, it has also been revealed that IL-10 is an independent outcome predictor in disease severity scores [36].

A large number of studies have investigated the roles and behaviors of these mediators in the pathogenesis of trauma, its severity and outcomes; however, to the best of our knowledge, there are no data on the association between these circulatory cytokine (IL-6, IL-1β, TNF-α, and IL-10) levels and nutritional status in patients with traumatic head injury. In this regard, the aim of the present study was to examine the relationship between serum levels of pro-inflammatory cytokines (IL-6, IL-1β, and TNF-α), anti-inflammatory cytokines (IL-10) measured once at baseline with changes in the nutritional status of patients with traumatic head injury (THI) assessed at three consecutive times (within 24 h of admission, day 6 and day 13) during their stays in the intensive care unit (ICU).

## 2. Materials and Methods

The present investigation is a prospective cohort study. The study sample consisted of 64 patients with THI with (GCS) scores less than 10 who were hospitalized in the (ICU) of the General Teaching Hospital in Sulaimani, the Kurdistan Region of Iraq from 20 November 2017 to 7 August 2018. Patients under 18 and over 65 years of age and those with previous chronic diseases such as diabetes, hypertension, chronic obstructive air diseases, heart diseases, malignancy, liver diseases, and a history of taking anti-platelet medications were excluded from the study.

The NUTRIC score (Nutrition Assessment in the Critically Ill) was utilized to screen for the risk of malnutrition within 24 h of admission and then on days 6 and 13 of the stay in the ICU. The nutritional status of the patient was assessed using the actual body weight of the patient measured with a bed scale (Balas digital body scale, Tehran, Iran) and body composition analysis (fat and lean body mass) measured with bioelectrical impedance analysis (BIA) (Body stat’s, London, UK), which was conducted within 24 h of admission and on days 6 and 13 of the stay in the ICU. In addition, mid-upper arm circumference (MUAC) was measured with a flexible measuring tape that was wrapped around the mid-upper arm within 24 h of admission and on days 6 and 13 of the stay in the ICU. Mid-upper arm circumference is a useful tool for a fast assessment of nutritional status, and it has been widely used as a nutritional tool in ill patients. Kubrak and Jensen (2007) revealed that MUAC is a simple indicator of nutritional status in acute care patients [2]. The thickness of the adductor pollicis muscle (APM) was also measured using a Lange caliper (UK) on both hands of the THI patients within 24 h of admission and on days 6 and 13 of the stay in the ICU. The thickness of the APM can be used to estimate the loss of muscle and is correlated with other anthropometrics. Recently, Caporossi et al. suggested that the thickness of the adductor pollicis muscle (TAPM) may be used to assess the nutritional status and predict the mortality of critically ill patients [37].

The Acute Physiology and Chronic Health Evaluation (APACHE II) scoring system and Sequential Organ Failure Assessment (SOFA) score were used to assess the severity of the disease and incidence of organ dysfunction. The APACHE II score is used worldwide to measure ICU performance [38,39]. The SOFA score is an integer ranging from 0 to 24, where a greater value corresponds to worse organ function. It has a relatively high specificity and sensitivity for predicting the prognosis in critically ill patients [40,41]. The APACHE II and SOFA scores were obtained within 24 h after admission and on days 6 and 13 of the stay in the ICU.

To determine the concentrations of serum cytokines (IL-6 ng/L, IL-1β pg/mL, TNF-α ng/L, and IL-10 pg/mL), blood samples were collected from THI patients within 24 h of their admission to the ICU. In total, 5 cc of blood was collected from each patient in the morning, and then the blood samples were placed on ice and centrifuged at 3000× *g*. The aliquoted serum was frozen at −70 °C. After the blood samples were obtained from the 64 THI patients, the immune assays were performed according to the manufacturer’s instructions with multiplexing technology (fully automated or semiautomated benchtop analyzer supplied by Shanghai Korain Biotech Co. Ltd., Shanghai, China) with specialized lab supplies from the enzyme-linked immunosorbent assay (ELISA) kit, antibodies, and proteins intended for life science research (intraassay CV < 8% and inter-assay CV < 10%).

To take the ethical considerations into account in the current study, it was carried out according to the standard clinical ethics guidelines, and ethical approval was obtained from the ethics committee of Tehran University of Medical Science, International Campus, Research Affairs under the code IR.TUMS.VCR.REC.1396.2676 dated 19th June 2017. Moreover, in the current study, we invited the patients or next to kin of the patients to participate in this research. Information about the aim and the process of study was provided on the consent form, and they were asked not to make an immediate decision. In addition, they had the right to ask questions regarding the study of the research team and to consult with anyone before signing the consent form, to make sure that they understand all the information on the consent form, answer all their questions, and ensure they understood their freedom to quit the study.

### Statistical Analysis

The patients’ sociodemographic data, including age, BMI, fat body mass, LBM, and laboratory measurements, were collected and are expressed as the means ± SD. The Kolmogorov-Smirnov test was conducted to determine the normality of the distributions of the collected data. The nonparametric data are expressed as the medians (interquartile ranges) (IQRs).

Given the lack of reference range for cytokines, median was adopted as the cut-off points, with the values lower and above the medians named low and high here, respectively. The median of IL-6, IL-10, IL-1β, and TNFα were 214, 367.3, 3482.7, and 505.6, respectively. The analysis carried out using these cutoffs. The serum concentrations of the cytokines were compared for parametric outcome measurements (BMI, FBM and LBM) using repeated measures analysis of variance. In addition, the serum concentrations of the cytokines were compared for MUAC, APM, APACHE II, SOFA, and NUTRIC scores using the non-parametric Friedman test, as these outcomes were not normally distributed. The level of statistical significance was set at *p* < 0.05. Data analysis was carried out with SPSS version 22.0 (SPSS Inc. Chicago, IL, USA).

## 3. Results

In the present cohort study, 64 patients with THI were recruited; there were 26 women and 38 men. They were aged between 20 and 64 years, with a mean age of 35.97 ± 11.5. Most of the patients (57%) lived in the city, while 42% resided outside of the city. In addition, most of the patients (81.2%) had a low risk of malnutrition at admission. The medians (IQRs) of their MUAC and APM measurements at admission were 27.2 (3) and 21 (5), respectively. The patients’ Mean ± SD values of BMI, FBM, and LBM at admission were 28.01 ± 3.51, 26.9 ± 6.35, and 48.2 ± 7.04, respectively. Moreover, the patients’ mean SOFA and APACHE II scores at admission were 11 ± 3.57 and 16.17 ± 5.07, respectively, see Table 1.

There were statistically significant decreases in BMI and LBM over time spent in ICU in patients with high levels of the cytokines studied, but not in those with low cytokine levels, except for IL-10 and IL-1β (BMI *p* = 0.02, 0.03, LBM *p* = 0.009, 0.008). There were significant decreases over time in FBM for patients with low and with high cytokine levels (*p* < 0.001 to *p* = 0.002), (Table 2).

Patients with low levels of cytokines (IL-6 and IL1β) at admission were shown to have significant improved mid-upper arm circumference MUAC (*p* < 0.001 to 0.005), except for the low level of IL-10 and TNFα (*p* = 0.607, 0.653) respectively, and the low level of cytokines was not significant with APM “Table 3”. In contrast, patients with high levels of the studied cytokines tend to have significantly reduced mid-upper arm circumference (MUAC) and abductor pollicis muscle (APM) thickness (*p* < 0.001), (Table 3).

The analysis also showed that among patients with high levels of the studied cytokines there were significant associations with the THI patients’ APACHE II and SOFA scores and time in ICU. THI patients with high levels of the studied cytokines had a significantly worse clinical outcome (*p* < 0.001). However, THI patients with low levels of the studied cytokines had significantly clinical improvement (*p* < 0.001 to 0.034), (Table 4).

The results of the current study also found there were significant relationships in THI patient stay in ICU and the NUTRIC score for patients with high level of studied cytokines. THI patients with high levels of the studied cytokines had a significantly higher risk of malnutrition (*p* < 0.001 to 0.01). Conversely, THI patients with low levels of cytokines had lower risk of malnutrition during their stay in the ICU, IL-10 and TNFα (*p* < 0.001 to 0.01), IL-6 and IL-1β (*p* 0.23 to 0.900), (Table 5).

## 4. Discussion

The present study is the first investigation into the relationships between the serum levels of pro-inflammatory cytokines (IL-6, IL-1β, and TNF-α) and anti-inflammatory cytokines (IL-10) measured once at the baseline with changes in the nutritional status of THI patients assessed at three consecutive times (24 h after admission, day 6 and day 13) during the hospital stay in the ICU. Therefore, it is impossible to discuss a number of its findings in relation to those reported by previously conducted studies.

As revealed by the results of the present study, Patients with a high level of cytokines showed significant changes in their BMI, FBM and LBM over the course of time during their ICU stay from the baseline until day thirteen. patients with low serum levels of the studied cytokines did not have a significant association with change in BMI or LBM and time spent in ICU, except for IL10 and IL-1β. Patients with low levels of cytokines did not show significant changes in their BMI over time during their stay in the ICU for IL6 and IL-1β; for FBM, however, the association was significant. No similar findings have been reported by previously conducted studies with regard to the association between pro- and anti-inflammatory cytokine levels and BMI, LBM and FBM among ICU-admitted patients with traumatic head injury. No similar findings have been reported by previously conducted studies with regard to the association between pro- and anti-inflammatory cytokine levels and BMI, LBM and FBM among ICU-admitted patients with traumatic head injury. A retrospective cross-sectional study was performed by Masha et al. (2016) to examine the nutritional status of THI patients and to detect changes in nutritional status from admission and over the course of seven days in the ICU. No significant changes in weight or BMI were observed among TBI patients over the course of seven days in the ICU [42]. However, Kim & Choi-Kwon (2011) [43] found a significant reduction in BMI of TBI patients during their stay in the ICU, and they observed this reduction particularly among malnourished patients. In addition, severe TBI patients showed significant decreases in weight due to hypercatabolic and hypermetabolic rates [44]. Hejazi et al. (2016) [45] found a significant decrease in body cell mass and the LBM of patients in the ICU on the discharge day, although they did not observe a change in fat mass. Moreover, a reduction in lean mass and an increase in fat mass was observed in the Fuentes et al. (2009) [46] study during the first seven days after admission to the ICU using the bioelectrical impedance method. It was found that the possible reason behind the decrease in the lean mass in trauma patients during their stay in the ICU is an increase in the level of inflammation because it assists in accelerating the degradation of muscle proteins [47]. A recent study found that patients with TBI and stroke lose weight in the acute phase after injury, and those with TBI in particular lose more weight than patients with stroke; patients with TBI are at higher risk of malnutrition [44]. Allard et al. (2015) [48] studied 373 patients who had their weight and nutritional status assessed using the Subjective Global Assessment (SGA) tool at admission. Twenty-five percent of the patients experienced weight loss of ≥5%, which was defined as nutritional deterioration. Fifty-one percent of them were classified as having SGA deterioration. It was noted that the nutritional status of ICU patients deteriorated in adequately nourished patients and in patients who were not adequately fed. This indicates that there might be other factors contributing to the deterioration of the trauma patients during their stay in the ICU [43]. However, in previous studies, changes in the nutritional status and cytokine levels were not studied. Therefore, this is the first study to observe the nutritional changes over the course of the time from the baseline to day 13 of THI patients during their stay in the ICU and to link these nutritional changes to the chief mediators of the acute phase response, namely, cytokines. The associations between cytokines and chronic diseases have been established in many studies. For example, Eagan et al., (2012) [49] indicated that the LBM in cachectic COPD patients had a significant relationship with TNF-α but not with IL-1β or IL-6. In addition, a significant correlation was reported between IL-6 and worse survival in metastatic breast cancer patients [50] and between BMI and IL-6 levels in patients with type-2 diabetes [51]. In contrast, a large number of studies have examined the role of nutritional support in traumatic brain injury patients. For instance, in an animal TBI study, oligonucleotide-based therapies produced some promising results. Oligomeric diets demonstrated potential benefits in rat models of TBI, preventing TBI-induced weight loss and thymus atrophy and, by extension, averting immune dysfunction [52].

Another finding of the present study was that low levels of the studied cytokines except IL-10 were significantly related to MUAC in THI patients. Mid-upper arm circumference of the THI patients significantly improved over the course of time from admission to day 7 and until day 13 for those patients with low levels of the studied cytokines (IL-6, IL-1β, and TNFα). In addition, this improvement was also observed for IL-10, but it was not significant. The low serum levels of all studied cytokines were related to APM in THI patients, but the relationships were not significant. The thickness of the abductor pollicis muscles of the THI patients improved over the course of time from admission to day 7 and until day 13 for those patients with low levels of the studied cytokines. However, high serum levels of the studied cytokines were significantly related to deterioration of the MUAC and APM in THI patients. Mid-upper arm circumference and the thickness of the abductor pollicis muscles of the THI patients significantly deteriorated over the course of time from admission to day 7 and until day 13 for those patients with high levels of the studied cytokines. These findings are in line with those of the study carried out by Hejazi et al., (2016), who observed that the mean MUAC and weight of patients were significantly reduced during their stay in the ICU [45]. In addition, our results are also in line with those of Nematy et al., (2011) [53], who indicated a significant reduction in anthropometric measurement among their patients in the ICU. A deterioration of anthropometric measurements was also observed in the Huang et al. study of patients staying in the ICU [54]; moreover, Sungurtekin et al., (2008) [55] observed lower MUAC, body mass index and weight in the malnourished patients compared to the well-nourished patients staying in the ICU. However, the mentioned studies did not link the deterioration of the anthropometric measurements of patients staying in the ICU to the level of inflammation in terms of the cytokines. Therefore, the novelty of the current study was linking the deterioration of the anthropometric measurements of patients staying in the ICU to the serum levels of cytokines. In surgical patients, APM thickness was found to be a good measurement of malnutrition and muscle depletion [56]. A study found a significant correlation between the thickness of the APM and anthropometric measurements of patients in the ICU [57]. However, Pereira et al. (2018) [58] demonstrated that the thickness of the APM was not a satisfactory method for detecting malnutrition in surgical patients in the ICU, although they observed that a low thickness of the APM was related to a prolonged hospital stay. The MUAC and APM measurements are two anthropometric measurements that reflect the patient’s body composition in terms of fat and muscle mass. The reason behind the deterioration of muscle mass is that TBI patients develop metabolic changes post-injury. For instance, the mean energy expenditure was found to be increased from 87% to 200% above the normal range for patients with TBI, and this increase might continue for 30 days after ICU admission [9]. In addition, high levels of the production of inflammatory mediators and corticosteroid hormones are considered the main factors responsible for the high metabolic rate [59,60]. Moreover, the hypercatabolic status of such patients predisposes the TBI patents to a negative nitrogen balance and in turn to losses in muscle mass [61]. This is the current interesting finding: THI patients with high levels of the studied cytokines were more prone to developing low nutritional status in terms of MAUC and the thickness of the APM during their ICU stay.

According to the analysis of the data, there were significant relationships between the studied cytokines and the APACHE II and SOFA scores among the THI patients. Patients with high serum levels of cytokines tend to have significantly worse clinical outcomes in terms of the severity of the disease as determined by the APACHE II score and more organ dysfunction as expressed by the SOFA score. This finding is in agreement with those of the studies conducted by Heyland et al., (2011) [62] and Lew et al., (2017) [63]. Therefore, the studied cytokines can be considered reliable indices for assessing the severity of the disease and organ dysfunction in THI patients, which can also be determined and assessed through the APACHE II and SOFA scores, respectively.

The last finding of the present study was the significant relationships between the high levels of the studied cytokines and NUTRIC score. THI patients with high serum levels of the studied cytokines tended to be more at risk of malnutrition over the course of time during their stay in the ICU. In contrast, patients with normal and low levels of the studied cytokines tend to be significantly less at risk of malnutrition during their stay in the ICU. The NUTRIC score was developed by Heyland et al., (2011) [62] to assist those patients staying in the ICU who might benefit from receiving nutritional therapy. One of the components of this tool is determining the concentration of IL6. Therefore, we are in line with previous findings, as high levels of Il6 are related to worse clinical outcomes in postoperative patients. We suggest including the other cytokines in the score as well in future studies.

Like any other study, the present experiment had some limitations. The first limitation was measuring the cytokines only at the time of admission to the ICU. Measuring their levels at subsequent stages in the ICU could provide a deeper understanding of their correlations with nutritional status and disease severity; therefore, future studies are recommended to take this point into account. The second limitation of the present study was related to the limited number of cytokines that were studied. If a larger number of pro- and anti-inflammatory cytokines are assessed, the nutritional status of traumatic patients can be predicted based on stronger evidence; therefore, future studies should include a larger number of cytokines and investigate their roles in the nutritional status of THI patients.

## 5. Conclusions

According to the findings of the present study, there were significant associations between changes in nutritional status and time spent in ICU when classifying patients by the serum concentrations of pro- and anti-inflammatory cytokines. Patients with high levels of pro- and anti-inflammatory cytokines had a significantly worse nutritional (BMI, LBM, FBM, MUAC and APM). In addition, patients with high levels of the pro- and anti-inflammatory cytokines had significantly worse clinical progress. Moreover, patients with high levels of the pro- and anti-inflammatory cytokines were significantly more prone to be at risk of developing malnutrition.

## Figures and Tables

**Table 1:** The patients’ baseline characteristics (*n =* 64).

Variables	No. (%)
Age (Mean ± SD)/years	35.9 ± 11
Age range/years	20–64
Sex	
Male	38 (59.4)
Female	26 (40.6)
Address	
Inside city	37 (57.8)
Outside city	27 (42.2)
NUTRIC score at admission	
Low malnutrition risk	52 (81.2)
High malnutrition risk	12 (18.8)
BMI kg/m^2^	
(Mean ± SD)	28.01±3.51
MUAC/cm	
Median (IQR)	27.2 (3)
APM/cm	
Median (IQR)	21 (5)
FBM/kg	
(Mean±SD)	26.9 ± 6.35
LBM/kg	
(Mean±SD)	48.2 ± 7.04
APACHE II score	
Median (IQR)	18.0 (10)
SOFA score	
Median (IQR)	12 (5)

**Table 2:** The associations between serum concentration levels of pro- and anti-inflammatory cytokines and nutritional status.

Measurements		BMI KG/M^2^			^#^*p*-Value	FBM			*p*-Value	LBM			# *p*-Value
		24 h after Admission Mean ± SD	At Day 6 Mean ± SD	At Day 13 Mean ± SD		24 h after Admission Mean ± SD	At Day 6 Mean ± SD	At Day 13 Mean ± SD		24 h after Admission Mean ± SD	At Day 6 Mean ± SD	At Day 13 Mean ± SD	
IL-6 ng/L	Low level (*n =* 32)	27.66 ± 3.91	27.55 ± 4.00	27.58 ± 3.95	0.52	24.97 ± 6.08	24.55 ± 6.04	24.52 ± 5.86	0.002	50.47 ± 6.49	50.03 ± 6.40	49.61 ± 6.00	0.08
	High level (*n =* 32)	28.33 ± 3.12	27.22 ± 3.03	26.59 ± 2.99	<0.001	28.76 ± 6.14	27.18 ± 5.84	25.67 ± 5.83	<0.001	46.18 ± 7.00	44.42 ± 6.75	43.66 ± 6.45	<0.001
IL-10 pg/mL	Low level (*n =* 32)	28.47 ± 4.03	27.99 ± 4.16	27.87 ± 4.13	0.02	26.70 ± 6.98	25.91 ± 6.62	25.70 ± 6.49	0.001	48.68 ± 6.57	47.69 ± 6.84	47.31 ± 6.37	0.009
	High level (*n =* 32)	27.51 ± 2.85	26.73 ± 2.55	26.22 ± 2.45	<0.001	27.16 ± 5.72	25.90 ± 5.45	24.48 ± 5.05	<0.001	47.81 ± 7.59	46.54 ± 7.47	45.73 ± 7.39	<0.001
IL-1β pg/L	Low level (*n =* 32)	27.98 ± 3.94	27.82 ± 3.99	27.77 ± 3.94	0.13	25.71 ± 6.17	25.19 ± 6.02	25.00 ± 5.82	<0.001	50.65 ± 6.50	49.79 ± 6.73	49.78 ± 5.98	0.051
	High level (*n =* 32)	28.03 ± 3.12	26.97 ± 2.99	26.41 ± 2.93	<0.001	28.06 ± 6.41	26.58 ± 6.07	25.21 ± 5.91	<0.001	46.02 ± 6.79	44.64 ± 6.63	43.74 ± 6.54	<0.001
TNF ng/L	Low level (*n =* 32)	28.57 ± 4.24	28.15 ± 4.12	28.03 ± 4.03	0.03	27.03 ± 7.14	26.18 ± 6.76	25.88 ± 6.62	0.001	49.21 ± 6.53	48.11 ± 6.79	47.75 ± 6.19	0.008
	High level (*n* = 32)	27.41 ± 2.47	26.56 ± 2.52	26.05 ± 2.49	<0.001	26.81 ± 5.52	25.61 ± 5.25	24.29 ± 4.82	<0.001	47.25 ± 7.53	46.09 ± 7.42	45.26 ± 7.41	<0.001

^#^*p*-value was determined using repeated measures analysis of variance. Abbreviations: BMI, body mass index; FBM, fat body mass; LBM, lean body mass; SD, standard deviation.

**Table 3:** The associations between serum concentration levels of pro- and anti-inflammatory cytokines and some nutritional parameters.

Measurements		MUAC			* *p*-Value	APM			* *p*-Value
		24 h after Admission Mean (IQR)	At Day 6 Mean (IQR)	At Day 13 Mean (IQR)		24 h after Admission Mean (IQR)	At Day 6 Mean (IQR)	At Day 13 Mean (IQR)	
IL-6 ng/L	Low level (*n* = 32)	26.57(3.93)	26.94 (4.50)	27.17 (3.19)	<0.001	21.13 (5.00)	21.14 (5.00)	21.20 (4.80)	0.344
	High level (*n* = 32)	28.05(2.60)	26.93 (2.95)	25.77 (3.11)	<0.001	21.42 (4.50)	20.01 (4.55)	19.46 (5.05)	<0.001
IL-10 pg/mL	Low level (*n* = 32)	27.73 (3.65)	27.53 (3.97)	27.46 (3.85)	0.607	22.08 (4.45)	21.73 (4.65)	21.52 (5.05)	0.075
	High level (*n* = 32)	26.87 (2.80)	26.30 (2.10)	25.42 (2.60)	<0.001	20.43 (5.30)	19.31 (5.10)	19.00 (5.20)	<0.001
IL-1β pg/L	Low level (*n* = 32)	27.32 (3.48)	27.52 (3.75)	27.59 (3.45)	0.005	21.70 (4.60)	21.57 (4.00)	21.48 (4.70)	0.930
	High level (*n* = 32)	27.31 (2.95)	26.36 (2.15)	25.38 (2.45)	<0.001	20.89 (5.45)	19.60 (5.10)	19.19 (5.20)	<0.001
TNFα ng/L	Low level (*n* = 32)	27.80 (4.50)	27.60 (4.37)	27.54 (4.25)	0.653	22.15 (5.10)	21.66 (4.65)	21.72 (5.10)	0.23
	High level (*n* = 32)	26.80 (2.70)	26.23 (2.01)	25.34 (2.50)	<0.001	20.35 (4.50)	19.38 (5.10)	18.80 (5.20)	<0.001

* *p*-value was determined using fried man test for nonparametric variables; Abbreviations: MUAC, mid-upper arm circumference; APM, abductor pollicis muscle, IQR, interquartile range.

**Table 4:** The associations between serum concentrations of pro- and anti-inflammatory cytokines and outcomes of THI patients.

Measurements		APACHE II Score			* *p*-Value	SOFA Score			* *p*-Value
		24 h after Admission Mean (IQR)	At Day 6 Mean (IQR)	At Day 13 Mean (IQR)		24 h after Admission Mean (IQR)	At day 6 Mean (IQR)	At day 13 Mean (IQR)	
IL-6 ng/L	Low level (*n* = 32)	16.63 (12.00)	12.28 (4.00)	14.28 (4.00)	<0.001	10.06 (5.00)	8.59 (4.00)	9.91 (3.00)	<0.001
	High level (*n* = 32)	20.63 (6.00)	24.19 (5.50)	24.19 (4.00)	<0.001	13.84 (4.00)	15.34 (4.00)	16.06 (4.00)	<0.001
IL-10 pg/mL	Low level (*n* = 32)	17.69 (7.50)	16.06 (8.50)	14.94 (5.00)	<0.001	11.38 (5.00)	10.28 (4.50)	9.56 (4.50)	<0.001
	High level (*n* = 32)	20.87 (8.00)	20.41 (8.00)	22.31 (7.00)	0.01	14.34 (4.00)	13.66 (7.00)	14.59 (7.00)	0.034
IL-1β pg/L	Low level (*n* = 32)	16.03 (5.00)	13.91 (4.00)	15.75 (4.00)	<0.001	10.50 (4..00)	9.06 (4.00)	9.81 (4.00)	<0.001
	High level (*n* = 32)	21.22 (7.00)	22.56 (6.00)	22.72 (5.50)	<0.001	14.09 (4.00)	14.88 (5.50)	15.47 (4.00)	<0.001
TNFα ng/L	Low level (*n* = 32)	17.41 (4)	15.81 (8.00)	14..88 (6.00)	<0.001	11.41 (5.00)	10.31 (5.00)	9.69 (4.50)	<0.001
	High level (*n* = 32)	21.06 (9.00)	20.66 (8.00)	22.38 (7.00)	0.01	14.22 (4.00)	13.63 (7.00)	14.56 (7.00)	0.035

* *p*-value was determined using Friedman test for nonparametric variables; Abbreviations: APACHE II, Acute Physiology and Chronic Health Evaluation; SOFA, sequential organ failure assessment; IQR, interquartile range.

**Table 5:** The associations between serum concentrations of pro- and anti-inflammatory cytokines and NUTRIC score.

Measurements		NUTRIC score			* *p*-Value
		24 h after Admission Mean (IQR)	At Day 6 Mean (IQR)	At Day 13 Mean (IQR)	
IL-6 ng/L	Low level (*n* = 32)	1.50 (1.00)	1.50 (1.00)	1.50 (1.00)	0.900
	High level (*n* = 32)	5.09 (1.00)	6.06 (1.00)	7.03 (1.00)	<0.001
IL-10 pg/mL	Low level (*n* = 32)	2.48 (3.00)	2.76 (4.00)	3.03 (5.00)	0.01
	High level (*n* = 32)	4.32 (4.00)	5.06 (5.00)	5.81 (6.00)	0.001
IL-1β pg/L	Low level (*n* = 32)	1.82 (1.00)	1.92 (1.00)	2.03 (1.00)	0.23
	High level (*n* = 32)	4.82 (0.5)	5.70 (0.50)	6.58 (0.50)	<0.001
TNFα ng/L	Low level (*n* = 32)	2.48 (3.00)	2.76 (4.00)	3.01 (5.00)	<0.001
	High level (*n* = 32)	4.32 (3.00)	5.06 (4.00)	5.81 (5.00)	0.01

* *p*-value was determined using Friedman test for nonparametric variables. Abbreviations: NUTRIC, Nutrition Risk in Critically ill; IQR, interquartile range.
